# Audiovisual incongruence differentially impacts left and right hemisphere sensorimotor oscillations: Potential applications to production

**DOI:** 10.1371/journal.pone.0258335

**Published:** 2021-10-07

**Authors:** David Jenson

**Affiliations:** Department of Speech and Hearing Sciences, Washington State University, Spokane, Washington, United States of America; University of New England, AUSTRALIA

## Abstract

Speech production gives rise to distinct auditory and somatosensory feedback signals which are dynamically integrated to enable online monitoring and error correction, though it remains unclear how the sensorimotor system supports the integration of these multimodal signals. Capitalizing on the parity of sensorimotor processes supporting perception and production, the current study employed the McGurk paradigm to induce multimodal sensory congruence/incongruence. EEG data from a cohort of 39 typical speakers were decomposed with independent component analysis to identify bilateral mu rhythms; indices of sensorimotor activity. Subsequent time-frequency analyses revealed bilateral patterns of event related desynchronization (ERD) across alpha and beta frequency ranges over the time course of perceptual events. Right mu activity was characterized by reduced ERD during all cases of audiovisual incongruence, while left mu activity was attenuated and protracted in McGurk trials eliciting sensory fusion. Results were interpreted to suggest distinct hemispheric contributions, with right hemisphere mu activity supporting a coarse incongruence detection process and left hemisphere mu activity reflecting a more granular level of analysis including phonological identification and incongruence resolution. Findings are also considered in regard to incongruence detection and resolution processes during production.

## 1. Introduction

During speech production, sensory feedback is integrated into feedforward motor commands to enable online error detection and fluent coarticulation at normal speech rates [1]. These notions have been explicitly outlined in computational models of speech production such as Directions Into Velocities of Articulator (DIVA; [2, 3]) and State Feedback Control (SFC; [4]), with their assertions well supported by the results of auditory perturbation studies demonstrating online adaptations to vocal output as a function of unexpected perturbations to auditory reafference [5, 6]. Similar results are also observed during physical (i.e., somatosensory) perturbation of the larynx during speech production [7, 8], demonstrating the sensitivity of speech motor control to multiple forms of sensory feedback. However, the influence of auditory and somatosensory feedback signals on speech motor control are frequently probed in isolation, a critical omission given that speech gives rise to multimodal sensory feedback signals which must be evaluated and compared against each other. A recent study by Smith et al. [9] probed the influence of convergent and divergent feedback signals on speech motor control, demonstrating dynamic patterns of adaptive behavior in response to unimodal and multimodal sensory feedback perturbations. However, it remains unclear how these multimodal feedback signals are integrated in the brain and how these integration processes influence sensorimotor activity supporting speech processing [10, 11]. This is a critical gap in knowledge given the proposed role of sensory feedback anomalies in sensorimotor-linked disorders such as stuttering [12, 13]. The goal of the current study is therefore to clarify how the integration of convergent and divergent sensory streams influences sensorimotor activity.

This study probes these integration processes by employing the McGurk paradigm [14], in which the pairing of mismatched auditory and visual stimuli (e.g., audio /ba/ with visual /ga/) leads to a fusion percept (in this example, /da/). The use of this perception-based task has several advantages making it well suited to probing the influence of discrepancy-resolution processes on sensorimotor activity. First, the congruence/divergence between auditory and visual stimulus components is controlled by experimenters, removing a potential source of noise in sensorimotor predictions elicited during speech production [15, 16]. Second, audiovisual fusion provides a salient marker that multimodal sensory integration has occurred, enabling observed differences to be more clearly interpreted as through the framework of sensory fusion rather than more general effects of multisensory processing [17, 18], a mirroring response [19], or a particular audiovisual pairing. Third, perceptual tasks elicit similar sensorimotor responses as those elicited during production [10, 20–22] such that findings hold relevance for production. Fourth, the elicitation of the McGurk effect has been previously associated with Analysis by Synthesis [23], providing a robust theoretical framework for the interpretation of observed neural activity.

Analysis by Synthesis is a Constructivist perspective proposing that perception arises from the interaction of bottom-up sensory processing and top-down guidance by prior knowledge (e.g., motoric representations) [24–26]. Specifically, a coarse sketch of the incoming stimulus is relayed to anterior motor regions by an inverse (i.e., sensory to motor) model for mapping onto an articulatory representation. A sensory representation of this motor-based hypothesis is then returned to posterior sensory regions by a forward (i.e., motor to sensory) model for validation against the full signal [25]. Confirmed hypotheses lead to stimulus identification, while mismatches lead to iterative hypothesis-test loops until the mismatch is resolved and the stimulus is identified [27]. As these dynamic internal modeling processes fluctuate in response to stimulus parameters [28–30] and task demands [31, 32], it is imperative to evaluate these sensorimotor interactions with high temporal precision.

To probe sensorimotor dynamics during speech perception, Jenson et al. [20] recorded the mu rhythm, an oscillatory marker of sensorimotor activity [33] commonly recorded over anterior sensorimotor regions [22, 34–38] from a typical cohort during the accurate discrimination of /ba/ /da/ syllable pairs. Time-frequency (ERSP) decomposition of mu oscillations into constituent alpha (~10 Hz; sensory) and beta (~20 Hz; motor) frequency bands revealed robust patterns of activity, with concurrent alpha and beta desynchronization (ERD; active processing) emerging following stimulus offset and persisting across the remainder of the trial. Based on the similarity of the observed patterns to those elicited during speech production [36, 39] and working memory [40, 41], findings were interpreted as evidence of covert rehearsal to support working memory maintenance. Specifically, alpha and beta ERD were considered evidence of the paired inverse and forward models, respectively [11] instantiating covert production [10]. However, working memory maintenance is necessarily preceded by an encoding phase, in which the sensory signal is mapped onto a phonological representation [42]. Critically, the Analysis by Synthesis processes under investigation in the current study are engaged only in the encoding stage, and it is therefore essential to disentangle the influence of encoding and maintenance processes on mu oscillations. However, this disambiguation was not possible based on the experimental paradigm of Jenson et al. [20].

In a follow-up study to resolve this ambiguity, Jenson et al. [30] evaluated the mu rhythm during the discrimination of un-degraded syllable pairs and the same syllable pairs degraded with noise masking and robust filtering [43, 44]. While concurrent alpha and beta ERD was observed following stimulus offset in all conditions, activity was weaker in degraded compared to non-degraded conditions, normalizing in the late stage of the trial epoch. This was interpreted through the framework of Analysis by Synthesis to suggest that degraded conditions are characterized by a prolonged encoding phase and a delayed maintenance stage of working memory processing. Specifically, the low fidelity of degraded stimuli required more iterations through the hypothesis-test-refine loop to be successfully mapped onto phonological representations prior to the engagement of covert rehearsal. While the results of Jenson et al. [30] demonstrate the sensitivity of mu oscillations to the internal modeling processes which map auditory signals onto phonological representations, it is critical to consider how these processes may unfold in the McGurk paradigm.

The McGurk paradigm consists of the concurrent presentation of either congruent or incongruent auditory and visual signals [14]. Interpretations of Jenson et al. [30] notwithstanding, the capacity for lip-reading [45] and visual speech identification [46, 47] may be interpreted to suggest that the initial articulatory hypotheses supporting Analysis by Synthesis may be generated by either auditory or visual signals. It may then be proposed that in the McGurk paradigm, both visual and auditory streams give rise to independent articulatory hypotheses, which must be reconciled to allow the extraction of a unified phonological representation during working memory encoding. This assertion is consistent with previous investigations of the McGurk paradigm which have proposed an initial discrepancy detection stage followed by later resolution/integration processes [48–50]. In congruent (i.e., audiovisual match) trials, minimal discrepancy is anticipated between initial visual-based and auditory-based articulatory hypotheses, allowing for rapid phonological encoding in working memory. In contrast, in incongruent (i.e., audiovisual mismatch) trials resulting in sensory fusion, greater discrepancy is anticipated between initial auditory-based and visual based articulatory hypotheses, necessitating multiple iterations through hypothesis-test-refine loops to resolve the discrepancy and resulting in a protracted encoding phase of working memory. For incongruent trials in which sensory fusion does not occur, a discrepancy in initial articulatory hypotheses cannot be assumed, and a prolonged encoding phase should not be anticipated. Given its sensitivity to working memory encoding demonstrated by Jenson et al. [30], mu activity is expected to inform regarding how the sensorimotor system supports the integration of discordant sensory signals onto a single phonological representation in the McGurk paradigm.

Based on the notion that incongruent trials will elicit a sensory mismatch requiring a protracted phonological encoding phase to resolve initial hypotheses discrepancy [29, 30], and that protracted phonological encoding is marked by weaker alpha and beta ERD [30], it is hypothesized that early post-stimulus mu activity will be weaker in mismatched trials leading to sensory fusion. In line with the notion that a discrepancy in initial articulatory hypotheses cannot be assumed in the absence of sensory fusion, it is further hypothesized that there will be no difference in mu activity between congruent trials and incongruent trials that do not result in sensory fusion. The results of the current study are expected to clarify how the sensorimotor system supports the integration of the multimodal sensory streams arising during speech perception and production. Findings additionally hold promise for describing the neural dynamics of multimodal feedback integration during production as well as clarifying the nature of the underlying compromise in sensorimotor-linked disorders.

## 2. Methods

### 2.1 Participants

39 native English speakers (mean age = 24.5 years; 34 female; 1 left handed) with no reported history of hearing impairment, attention deficit (i.e. ADD / ADHD), or cognitive/communicative disorder were recruited for the current study. Handedness dominance was evaluated with the Edinburgh Handedness Inventory [51]. This study was approved by the Washington State University Institutional Review Board (WSU-IRB; protocol #17179–003) and conducted in accord with the principles of the Declaration of Helsinki. All subjects provided written informed consent on a document approved by the WSU-IRB prior to study participation. Participants were compensated for their participation.

### 2.2 Stimuli

The stimuli for the current study consisted of all possible audiovisual pairings (9 total) of /ba/, /da/, and /ga/ spoken by two different female speakers. Stimulus pairings were generated from a subset of the raw auditory and visual signals available from the OLAVS stimulus repository [52], with the speakers selected on the basis of minimal head movement during speech production and the similarity in the time course of sound onset across speech tokens. Videos were recorded at 25 frames per second and presented in 1920 x 1080 resolution. Visual signals showed the speaker’s full face and shoulders, with speakers displaying neutral affect and wearing a black shirt on a black background. Stimuli were initiated by a static image of the speaker’s face with the mouth closed, which represented the last video frame prior to movement onset. Due to variability in speech movements across tokens and speakers, the duration of this static image varied from 280–640 ms. The next portion of each video consisted of the speaker’s facial movements as they articulated one of the speech tokens (/ba/, /da/, /ga/), with their mouth returning to the closed position following articulation. This final frame of speech movement in which the oral aperture was closed was presented as a static image for the remainder of the video. Due to variability in the timing of speech movements across speakers and tokens, this static image appeared between 1360 and 1640 ms from video onset. Auditory signals were recorded at 48 kHZ and consisted of the spoken production of one three speech tokens. To minimize the potential for differential stimulus processing based on the unconscious association of a given face/voice pairing [53], audiovisual signals were not combined across speakers. Subject to this constraint, 18 audiovisual pairings (2 speakers x 9 tokens) were generated for use in the current experiment, with all resulting videos being 2200 ms in length. Despite the variability in the timing of movement onset across speech tokens, it should be noted that trials were aligned by auditory onset and that movement onset for all stimuli falls outside the data analysis window. A pilot study confirmed both the perceptibility and the reliable elicitation of sensory fusion for the resulting stimuli.

### 2.3 Design

The experiment consisted of a three condition, within-subjects design. The three conditions presented to subjects are listed below, while the contribution of each audiovisual stimulus pairing to experimental conditions is shown in Table 1.

1. Audiovisual match; (Match)
2. Audio /ba/ paired with visual /ga/; (McGurk)
3. All other pairings of /ba/, /da/, or /ga/; (Mismatch)

**Table 1:** Contributions of audiovisual pairings to stimulus conditions.

Stimulus pairing^a^	Condition	Number of trials^b^
Aba_Vba	Match	40
Aba_Vda	Mismatch	12
Aba_Vga	McGurk	120
Ada_Vba	Mismatch	12
Ada_Vda	Match	40
Ada_Vga	Mismatch	12
Aga_Vba	Mismatch	12
Aga_Vda	Mismatch	12
Aga_Vga	Match	40

^a^Stimulus names represent the auditory and visual portions of each pairing (e.g., Aba_Vga corresponds to audio “ba” paired with visual “ga”).

^b^Stimuli were recorded from two different speakers, with each speaker’s tokens comprising half of the trials presented for each audiovisual pairing.

The Mismatch condition consisted of all possible mismatched audiovisual pairings (not including the McGurk pairing), which do not lead to sensory fusion. This condition was included as a distractor stimulus set to control for the presence of audiovisual mismatch [54], allowing neural differences observed during the perception of the McGurk effect to be interpreted as indices of sensory fusion. Stimulus presentation was fully randomized, with the ratio of stimuli predetermined, but the actual order of stimulus presentation randomly determined for each subject and experimental block by the presentation software. The proportion of stimuli per condition is based on the designs of Roa Romero et al. [49, 54], which served as models for the current study.

### 2.4 Procedures

The experiment was conducted in a double-walled, sound-treated booth fit with a faraday cage to minimize electromagnetic interference. Participants were seated in a comfortable chair with their head and neck well-supported. Stimulus presentation and acquisition of behavioral responses (via button press) was performed by a desktop computer running E-Prime version 3.0 coupled with a Brain Products TriggerBox to allow the timing of stimulus presentation to be marked in the EEG data stream. The auditory portions of stimuli were presented binaurally at 70 dB SPL with Etymotic insert earphones (ER1-14A). Visual signals were presented on a computer monitor approximately 57 inches / 145 cm from subjects, with the speaker’s face subtending a visual angle of approximately 11.03. The response cue for all trials was a 2x2 grid (mimicking the layout of buttons on the response pad) containing the options “Ba,” “Da,” “Ga,” and “Something Else” [55]. Subjects were instructed to press the button corresponding to the option that they heard on a Cedrus RB-844 response pad. Four permutations of the response grid with different arrangements of the options were used to ensure that subjects could not select a response prior to the end of the trial. Anticipatory motor planning can precede movement onset by up to 2000 ms [56], and it was necessary to ensure that sensorimotor activity associated with the button press did not contaminate activity associated with stimulus processing. While handedness of the button press response was not directly controlled, the response grid permutation was randomly selected on each trial, and subjects were instructed to use left and right thumbs and forefingers on the response pad. Consequently, there is no reason to suspect that handedness of behavioral responses influenced results.

**Fig 1:**
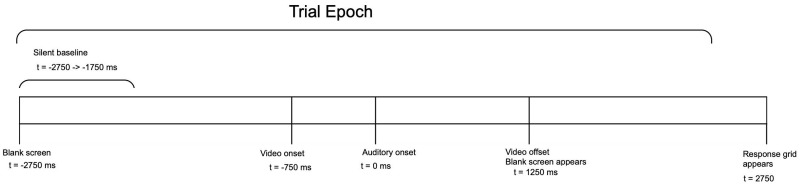
Epoch timeline. 4750 ms trial epoch timeline for single trials across all perceptual conditions.

All trial epochs were 4750 ms in length, ranging from -2750 ms to +2000 ms around time zero, defined as the onset of the auditory portion of stimuli. All epochs were initiated by a silent baseline of 1000 ms (i.e., -2750 to -1750 ms) taken from the inter-trial interval to enable subsequent ERSP decomposition. Following the baseline, the screen was blank (i.e., black) until ~750 ms prior to time zero (740–752 depending on the stimulus), at which point a static image of the speaker’s face (last frame before movement onset) was displayed. Time point zero was defined as the onset of the auditory portion of the stimulus. At +1250 ms, a blank screen was presented for 1500, after which the response grid was displayed until subjects indicated their selection via button press. Stimuli were presented in 4 blocks of 75 trials each (300 total), with the conditions randomly distributed within each block and separated post-hoc for the purpose of analysis. The timeline of trials is shown below (Fig 1).

### 2.5 Neural data acquisition

Whole head EEG data were recorded from two 32 channel actiCHamp active electrode modules (64 channels total) configured in an Easycap actiCAP according to the extended 10–20 system [57]. EEG data were acquired Using Brain Vision Recorder coupled with the Brain Products actiCHamp system. During signal acquisition, EEG data were band pass filtered (.016–250 Hz) and digitized at a sampling frequency of 1 kHz. Neural data from all four experimental blocks were captured in a single data file with the reference channel set to FCz.

### 2.6 Data processing

All processing of neural data was conducted in EEGLAB 14.1.2b [58, 59], an open-source Matlab (R2015b) toolbox for the analysis of electrophysiologic data. Data were processed at both individual and group levels to identify the mu rhythm and evaluate differences in time-frequency fluctuations across conditions, respectively. Each step of the data-processing pipeline listed here is discussed in greater detail below:

### 2.7 Processing pipeline

1. Individual Processing
  - Pre-processing of individual subjects’ raw data;
  - Independent Component Analysis (ICA) of individual data files to identify independent components of neural activity;
  - Source localization of all independent components per subject.
2. Group Processing
  - Pre-processed data files from each subject submitted to the EEGLAB STUDY module;
  - Independent components common across subjects identified via Principal Components Analysis (PCA);
  - Identification of bilateral mu clusters from the results of PCA;
  - Time frequency decomposition (via ERSP) of bilateral mu clusters;
  - Source localization of mu clusters with equivalent current dipole models.

### 2.8 Individual pre-processing

Raw data files for each subject were re-referenced to linked mastoid channels for the reduction of common mode noise and downsampled to 256 Hz to reduce the computational demands of subsequent processing steps. Data were then bandpass filtered from 3–35 Hz (-6 dB roll-off) to reduce the influence of muscle artifact and enable clear visualization of alpha and beta frequency bands. Next, 4750 ms epochs, ranging from -2750 -> +2000 ms around time zero (defined as auditory onset) were extracted from the continuous dataset (300 epochs total). Channel data were then visualized, with noisy channels identified based on the presence of either high frequency noise or large nonlinearities in the data. Channels deemed to be noisy at this stage were removed from the dataset. Additionally, to control for the potential influence of salt-bridging [60, 61], correlation coefficients were calculated for each pair of channels with custom Matlab code. Correlations exceeding 0.99 were considered evidence of salt-bridging [30]. For salt-bridged channel pairs, one of the channels was removed from the dataset to eliminate signal redundancy, a necessary step as all signals submitted to ICA must constitute independent observations [62].

In order to be retained for further analysis, data epochs had to meet three distinct criteria. First, the button press response had to indicate a ‘correct’ behavioral response. For Match trials, the behavioral response had to correspond to the identity of auditory and visual signals (i.e., “Ba,” “Da,” or “Ga”). For McGurk epochs (audio /ba/ paired with visual /ga/) to be retained, subjects had to respond either “Da” (fusion percept) or “Something Else,” indicating that the visual signal altered their perception of the auditory stimulus [55]. For Mismatch trials to be included, the behavioral response had to match either the auditory or visual component of the stimulus. Application of this inclusion criteria to Mismatch trials ensured that no integration of sensory signals occurred, enabling Mismatch trials to serve as a control on audiovisual mismatch for McGurk trials. Trials in which the behavioral responses did not meet the above criteria were removed from the dataset. Additionally, to confirm that subjects were actively attending to stimuli and engaged in the experimental task, behavioral responses had to occur within 2 seconds of the response cue for trial epochs to be retained. Finally, the remaining trial epochs were visually inspected and removed if they contained gross artifact. All remaining usable trials were then submitted to further steps of the processing pipeline. If behavioral responses for an individual subject indicated that they did not experience sensory fusion in at least 15% of McGurk trials [49], all data from that subject was removed from the study.

### 2.9 ICA

Pre-processed datasets for each subject were decorrelated with an extended Infomax algorithm [63] prior to ICA training using the “runica” algorithm. The initial learning weight was set to 0.001 and a specified stopping weight criterion of 10^−7^. The number of independent components (ICs) returned by ICA training conforms to the number of channels in the pre-processed dataset, with a maximum of 62 ICs per subjects (64 recording channels– 2 reference channels). However, the true number of ICs returned by ICA varied across subjects based on the differential number of channels rejected during pre-processing. Scalp maps, representing coarse estimates of the scalp distribution for each IC were generated by projecting the inverse weight matrix (W^-1^; resulting from ICA decomposition) back onto the original recording montage. To reduce the computational demands of subsequent processing steps, the resulting components were evaluated with the Multiple Artifact Rejection Algorithm (MARA), an open-source EEGLAB plug-in which employs machine learning to evaluate component likelihood of being artifactual [64]. Components deemed to have a 40% chance or higher of being artifact [65] were excluded from further stages of the processing pipeline.

### 2.10 Dipole localization

Each component identified by ICA was mapped onto an equivalent current dipole (ECD) model with the DIPFIT toolbox [66, 67], yielding point source estimates for the location of neural generators. Standardized 10–20 electrode coordinates for the recording montage were warped to a 4-shell spherical (i.e., BESA) head model, with automated coarse and fine fitting to the head model yielding single models for each of the 1581 components. The resultant dipole models constitute physiologically plausible solutions to the inverse problem and represent hypothesized generator sites for component activations. Dipole models were then back-projected onto the recording montage and compared against the original scalp recorded signal. The residual variance (RV%), constituting a measure of “goodness of fit” of the dipole models is determined by the mismatch between this back-projection and the original scalp-recorded signal, with lower RV% indicating a better ECD fit to the original signal.

### 2.11 STUDY module

All group level analyses were performed in the EEGLAB STUDY module, which allows IC activity to be evaluated across subjects and conditions. Processed data files from each subject were loaded into the STUDY modules, and all components localized within the cortical volume with RV < 20% were submitted for analysis. This threshold was selected as higher levels of variance likely indicate the presence of artifact or noise. While all experimental conditions were contained within a single dataset for each subject, distinct event markers were coded for each AV stimulus, allowing data from each condition to be segregated for analysis within the STUDY module.

### 2.12 PCA clustering

Component pre-clustering was performed on the basis of similarities in scalp maps, dipole localization, and component spectra across subjects. Principal Component Analysis (PCA) was implemented using the K-means statistical toolbox to group similar components based on specified criteria. Components were assigned to 38 clusters, from which bilateral mu clusters were identified on the basis of similarities in spectra, scalp maps, and dipole location. Final allocation of components to mu clusters was based primarily on the results of PCA, though all clusters were visually inspected to ensure that all components allocated to mu clusters met inclusion criteria, and no components meeting inclusion criteria had been mis-allocated. Inclusion criteria for mu clusters included a characteristic mu spectrum with peaks in alpha and beta frequency bands, RV < 20%, and ECD localization to accepted mu generator sites (i.e., Brodmann’s areas 1–4 or 6; [34]). Components determined to have been misallocated were then manually reassigned. To normalize the contribution of each subject to group-level analyses, only one component per subject was included in left and right mu clusters. For subjects contributing more than one component to each cluster, only the component with the lowest RV% was retained.

### 2.13 Source localization

Following final allocation of neural components to bilateral mu clusters, clusters were localized through ECD methods. The ECD source localization for each cluster represents the mean of the *(x*, *y*, *z)* Talairach coordinates for each contributing component. The resulting stereotactic coordinates were submitted to the Talairach Client for mapping to anatomic locations, yielding estimates of most likely source locations and associated Brodmann’s areas for mu clusters.

### 2.14 ERSP

ERSP analysis was performed to evaluate fluctuations in spectral power (in normalized dB units) between 3 and 35 Hz across the time course of perceptual events. Single trial data ranging from 3 to 35 Hz were decomposed with a family of Morlet wavelets, with 3 cycles at 3 Hz and an expansion factor of 0.8. ERSP data were referenced to a surrogate distribution generated from 200 time points randomly selected from a 1000 ms silent baseline extracted from the inter-trial interval [68]. This baseline window was selected to fully characterize sensorimotor responses to both auditory and visual portions of stimuli, as information from neither sensory stream was present during this time period. Individual ERSP changes were calculated with a bootstrap resampling method (*p* < .05, uncorrected). Statistical comparisons across conditions employed permutation statistics with cluster-based corrections for multiple comparisons [69].

## 3. Results

One subject was excluded from the study as they disclosed a previous closed-head injury after participating in the study. While the long-term impact of closed-head injuries on the electroencephalogram remains unclear [70], data from this subject was excluded out of an abundance of caution. Data from an additional 6 subjects were excluded from the analysis as they did not perceive the McGurk effect in at least 15% of trials [49]. Neural data from one additional subject was rendered unusable due to an equipment error. Consequently, data from only 31 participants were submitted for further analyses.

### 3.1 Behavioral data

Of the subjects who contributed to mu clusters, the mean number of trials in which the McGurk effect was perceived out of 120 presentations was 88.2 (SD = 26.5). However, the number of usable trials was less than this as two additional criteria (i.e., response latency < 2 seconds, devoid of artifact) had to be met for trial inclusion. The mean number of usable trials per condition for subjects contributing to mu clusters were: Match = 105 (SD = 9.4), McGurk = 78.1 (SD = 24.5), and Mismatch = 42.4 (SD = 9.72). A repeated measures ANOVA with Greenhouse-Geisser corrections [*ε* = .56] for violations of sphericity [X^2^ = 47.57, *p* < .001] revealed a main effect of Condition [F(1.11,32.04) = 109.24; *p* < .001]. Bonferroni-corrected post-hoc tests revealed that the mean number of usable trials differed across all conditions. Specifically, the number of trials in the Mismatch condition was less [*t*(29) = 6.48, *p* < .001] than the McGurk condition, which itself contained fewer [*t*(29) = 5.86, *p* < .001] trials than the Match condition.

### 3.2 Cluster characteristics

**Fig 2:**
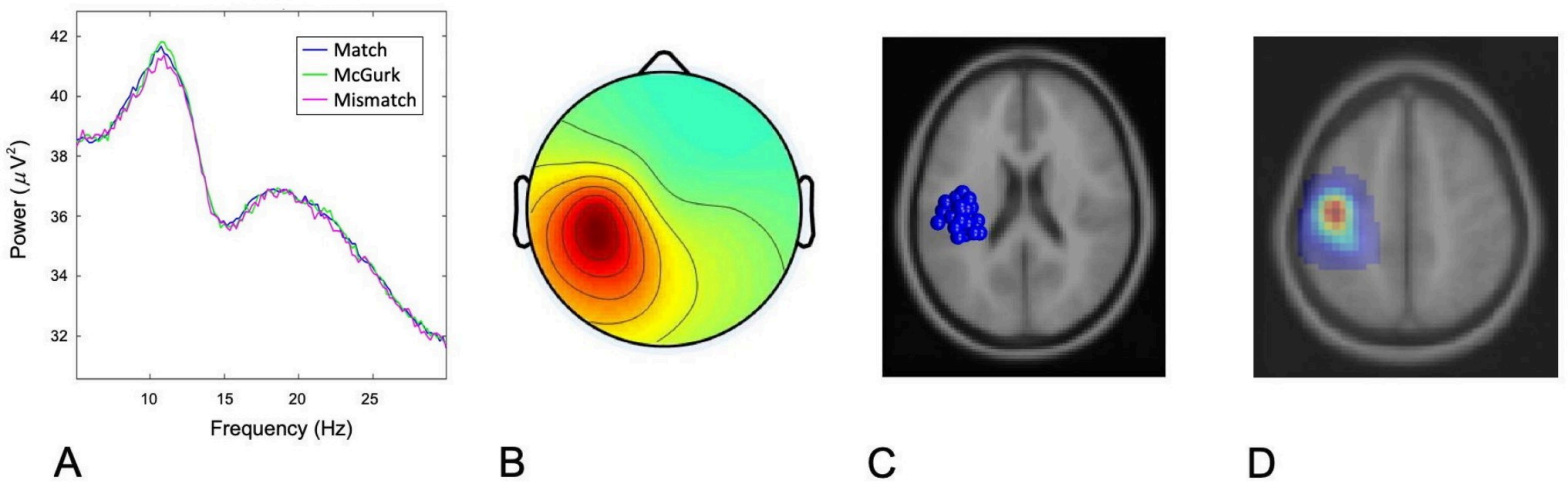
Cluster characteristics for left mu cluster. (A) Mean spectra for cluster components. (B) Mean scalp map for left hemisphere mu cluster. (C) ECD source localization estimates for contributing components. (D) Probabilistic dipole density, demonstrating maximal cluster localization.

**Fig 3:**
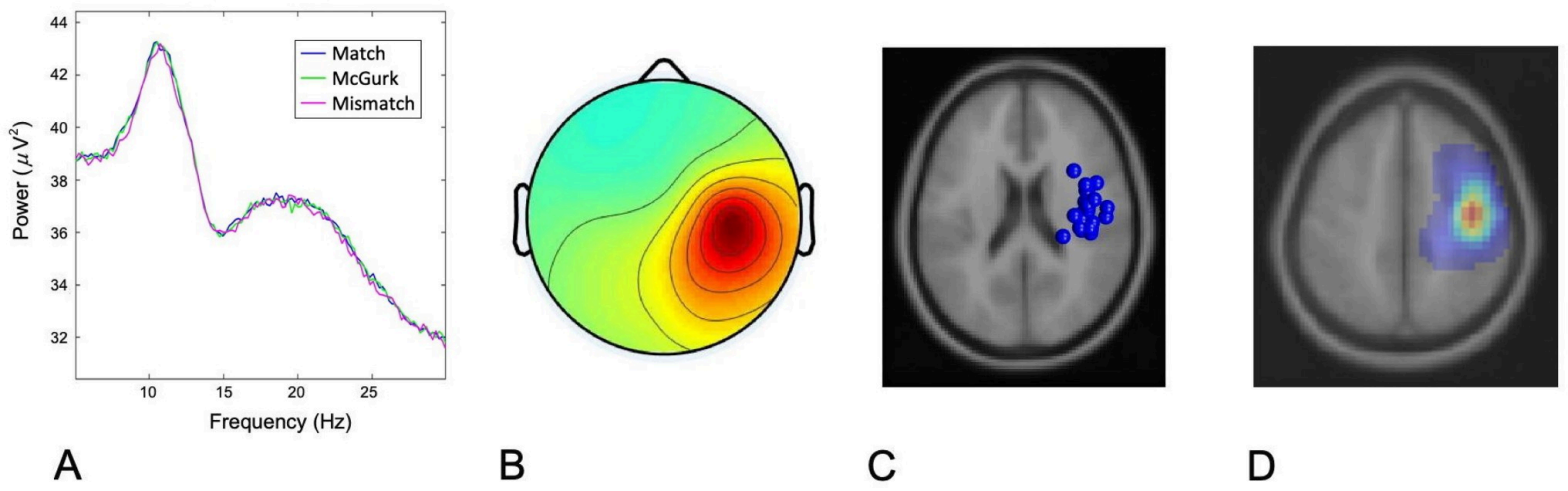
Cluster characteristics for right mu cluster. (A) Mean spectra for cluster components. (B) Mean scalp map for right hemisphere mu cluster. (C) ECD source localization estimates for contributing components. (D) Probabilistic dipole density, demonstrating maximal cluster localization.

The distribution of components contributing to left and right hemisphere mu clusters, respectively, are shown below (Figs 2 and 3). Of the subjects whose neural data was submitted for analysis, 29/31 contributed to the left hemisphere mu cluster and 28/31 contributed to the right hemisphere mu cluster. Specifically, 27 contributed components to both left and right mu clusters, 2 contributed to the left mu cluster only, and 1 contributed only to the right mu cluster. The mean ECD dipole localization for the left mu cluster was at Talairach (-40, -19, 46) in the postcentral gyrus (BA– 3) with residual variance of 1.89%. The mean dipole localization for the right mu cluster was at Talairach (40, -11, 47) in the precentral gyrus (BA– 4) with residual variance of 1.83%.

### 3.3 ERSP characteristics

#### 3.3.1 Left hemisphere

ERSP data from the left hemisphere mu cluster was characterized by weak alpha ERS (event related synchronization; inhibition) concurrent with the onset of the auditory signal, followed by the emergence of paired alpha and beta ERD at ~200 ms following acoustic onset in all conditions. Alpha ERD persisted across the remainder of the trial epoch in all conditions, while the time course of beta ERD was more variable. Specifically, beta ERD persisted across the remainder of the trial epoch in the McGurk condition, while ending ~850 ms following acoustic onset in both Match and Mismatch conditions. A 1 x 3 ANOVA employing permutation statistics with cluster-based corrections for multiple comparisons [69] identified omnibus alpha and beta differences spanning ~400–800 ms, and from 900 ms throughout the remainder of the trial epoch.

**Fig 4:**
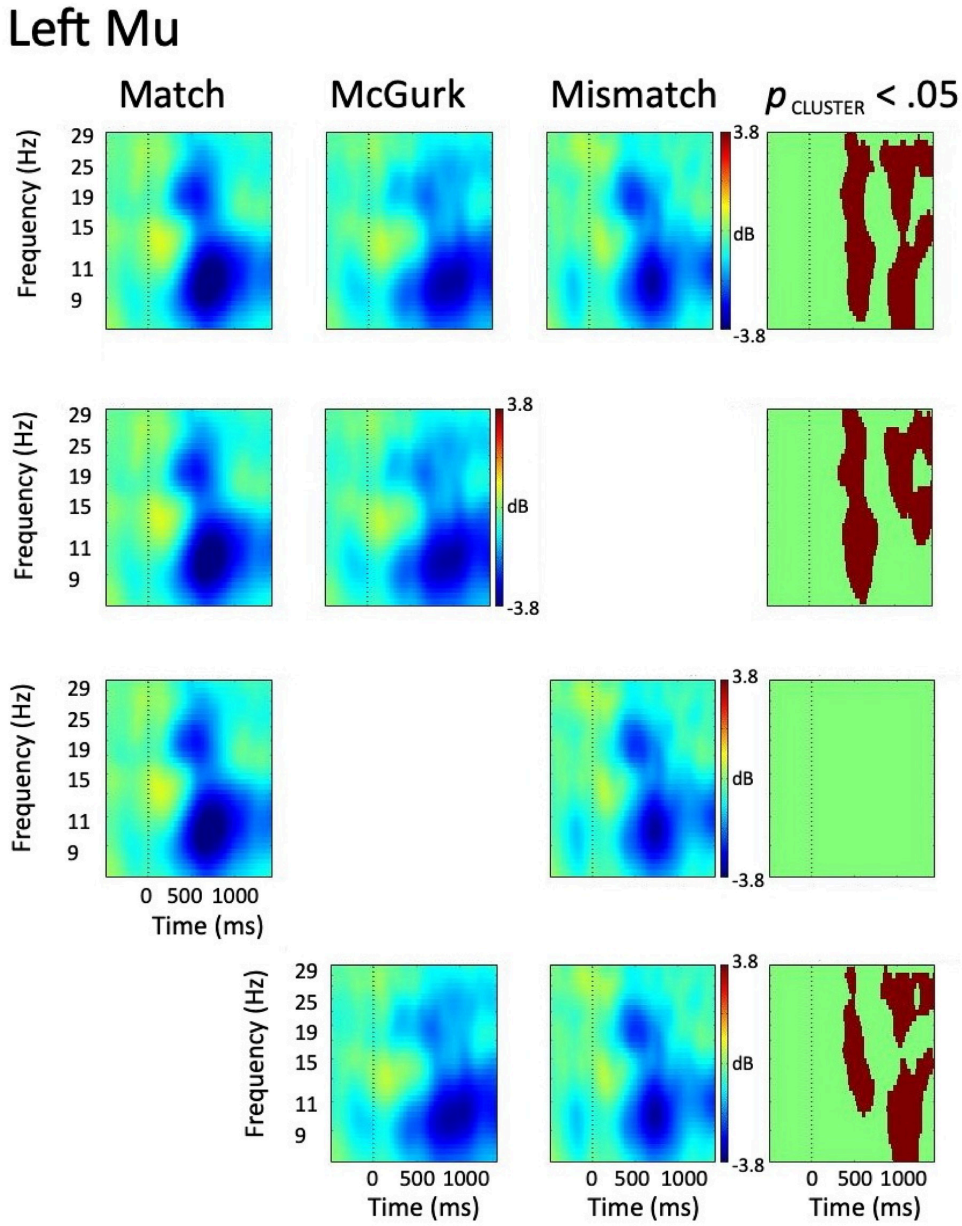
ERSP data from left hemisphere mu cluster. The first three columns represent data from Match, McGurk, and Mismatch conditions, with the right-most column showing time-frequency voxels significant at *p* < .05 (cluster corrected). The top row shows the results of the omnibus tests, while the lower rows show the results of the post-hoc tests.

To decompose the omnibus effect, a series of paired *t*-tests employing permutation statistics with cluster-based corrections for multiple comparisons were performed between each pair of conditions. The results of these post-hoc tests revealed no differences between Match and Mismatch conditions, while alpha and beta differences were observed between Match and McGurk conditions as well as between McGurk and Mismatch conditions. Alpha and beta differences were observed between Match and McGurk from ~300–800 ms and ~900 ms through the remainder of the trial epoch. Alpha and beta differences between McGurk and Mismatch conditions were observed from ~350–700 ms and ~800 ms through the remainder of the trial epoch. The results of the left hemisphere omnibus and post-hoc tests can be seen in Fig 4.

#### 3.3.2 Right hemisphere

ERSP data from the right hemisphere mu cluster was characterized by alpha ERS slightly preceding the onset of the acoustic signal, with paired alpha and beta ERD emerging following auditory onset in all conditions. Though the timeline of alpha ERD emergence varied across conditions (i.e., ~350 ms in the Match, ~500 ms in McGurk and Mismatch), alpha ERD persisted across the remainder of the trial epoch in all conditions. Beta ERD emerged ~300 ms following acoustic onset in Match and McGurk, though emerged slightly later (~450 ms) in the Mismatch condition. Following its emergence, beta ERD persisted throughout the remainder of the trial epoch in Match and McGurk trials, while it dissipated at ~1000 ms following acoustic onset in Mismatch trials. A 1 x 3 ANOVA employing permutation statistics with cluster-based corrections for multiple comparisons identified omnibus alpha differences from auditory onset through the remainder of the trial epoch, with beta differences spanning ~300–850 ms, and ~1000–1300 ms following acoustic onset.

**Fig 5:**
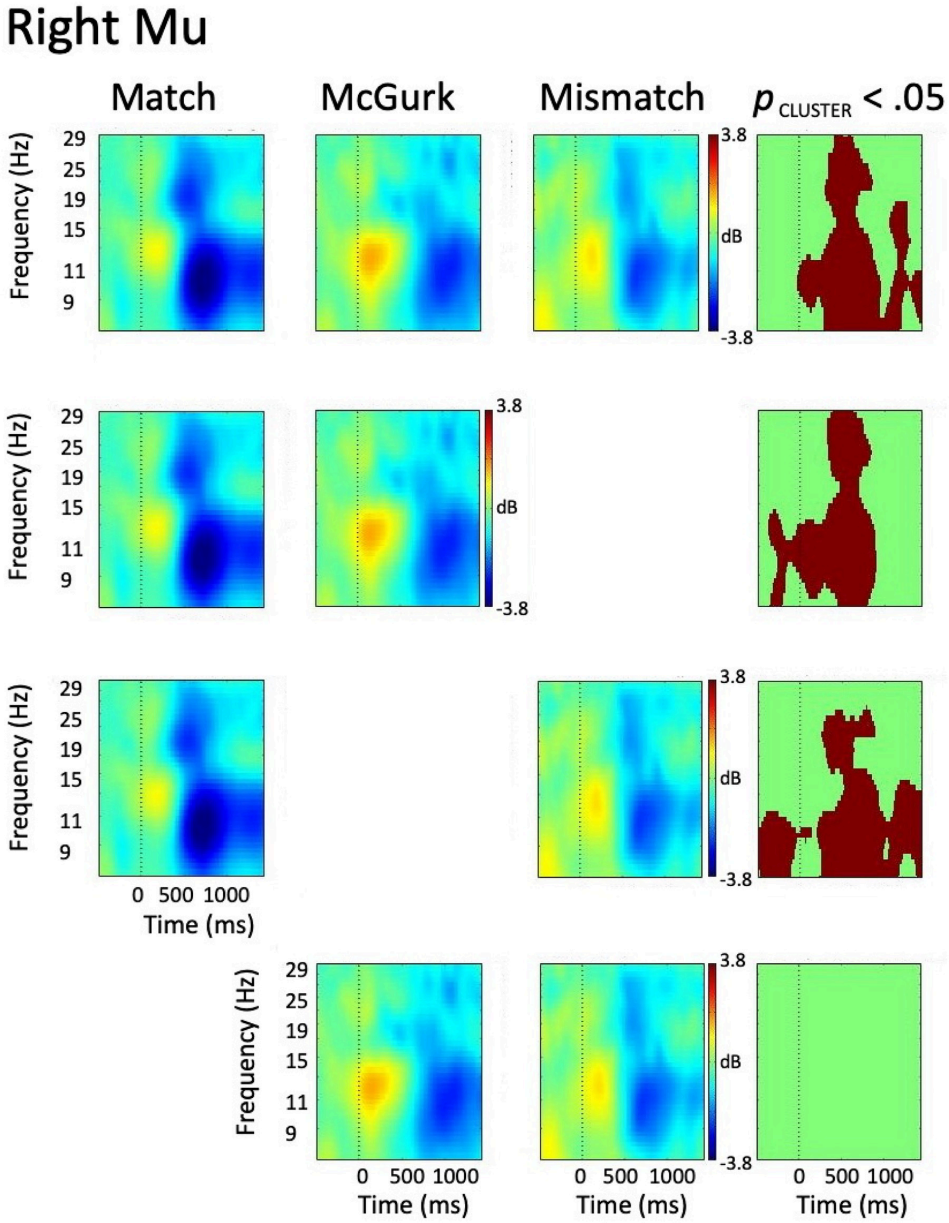
ERSP data from right hemisphere mu cluster. The first three columns represent data from Match, McGurk, and Mismatch conditions, with the right-most column showing time-frequency voxels significant at *p* < .05 (cluster corrected). The top row shows the results of the omnibus tests, while the lower rows show the results of the post-hoc tests.

To decompose the omnibus effect, a series of paired *t*-tests employing permutation statistics with cluster-based corrections for multiple comparisons were performed between each pair of conditions, with results indicating robust differences between condition pairs. The contrast between Match and McGurk revealed significant alpha differences from -400 ms to 900 ms, with stronger alpha ERS and weaker alpha ERD noted in the McGurk condition. Beta differences were observed from ~300–850 ms, with weaker beta ERD in McGurk compared to the Match condition. The comparison between Match and Mismatch revealed alpha differences throughout the trial epoch, with weaker alpha activity in Mismatch compared to Match. Beta differences, characterized by weaker beta ERD in Mismatch compared to Match, were present from ~250–850 ms. No significant differences were observed in the contrast between McGurk and Mismatch conditions. In order to minimize the potential for Type I error [71], consideration of post-hoc test results will be restricted to time-frequency voxels statistically significant in both the omnibus and post-hoc tests. While the overall patterns of activity appeared similar in both left and right hemispheres, patterns of between-condition differences were characteristically distinct. The results of the right hemisphere omnibus and post-hoc tests can be seen in Fig 5.

While it does not appear robust due to the color scale of Figs 4 and 5, it should be noted that weak bilateral ERD is present in alpha and beta bands prior to auditory onset in all conditions, though the magnitude of ERD following auditory onset is higher. This may be attributable to the variable time course of movement onset across the stimuli. Specifically, in contrast to auditory onset, which was temporally aligned across all trials, the onset of visual movement varied by ~350 ms across trials due to the variability in timing of speech movements across speakers and tokens. Alternatively, it may suggest stronger mu responses to multimodal than unimodal stimuli [72]. However, due to its weak appearance and the lack of a pure control condition against which its significance could be established, this pre-auditory mu activity is not considered further.

## 4. Discussion

Despite being a robust perceptual experience [73–75], the McGurk Effect is not perceived by all individuals [76], nor do those who perceive it do so reliably across all trials [77, 78]. In the current study, ~15% of subjects (6/39) were classified as non-perceivers and excluded from further analysis as they did not perceive sensory fusion in at least 15% of McGurk trials [49]. The proportion of subjects not susceptible to the McGurk illusion is not universally reported in the literature, nor is there a standardized threshold subjects must meet to be considered a ‘perceiver / non-perceiver.’ This consideration notwithstanding, reported ranges for the proportion of non-perceiving subjects range from 0–54% [48, 79] with an average of ~25% [54]. Among the subjects in the current study who reliably perceived the McGurk illusion, sensory fusion was reported in 73.5% of trials. While there is considerable variability across studies, published averages for the proportion of trials inducing the McGurk illusion range from 32–84% [48, 49, 80]. Given the reliable elicitation of the McGurk illusion in the current study, as well as the concordance with previous reports regarding both the proportion of perceiving subjects and trials eliciting sensory fusion, neural data are interpreted through the lens of the McGurk effect.

In the current study, ICA identified bilateral mu rhythms from a cohort of non-clinical control subjects during the perception of matched and mismatched audiovisual syllables. In line with other EEG studies employing similar inclusion criteria for cluster membership [81–84], 96% of McGurk-perceiving subjects contributed to mu clusters, with 93% contributing to the left mu cluster and 90% contributing to the right mu cluster. ECD models localized left and right mu clusters to BA-3 and BA-4, respectively, with activation spreading across anterior sensorimotor regions. It should be noted that while the localization of the left hemisphere cluster was slightly more posterior than in previous work from this lab, it is still consistent with accepted generator sites for mu rhythms [34, 85]. Consequently, given the high proportion of subjects contributing to mu clusters and the stability of mu spectra across conditions, it was possible to test experimental hypotheses regarding the integration of convergent and divergent sensory streams. However, before results from divergent (i.e., McGurk, Mismatch) trials can be meaningfully interpreted, it is first essential to consider how sensorimotor activity unfolds during convergent trials.

### 4.1 Overall patterns

In the Match condition, bilateral mu ERSP data was characterized by weak alpha ERS temporally aligned with the onset of auditory stimulation. Alpha ERS gradually transitioned to concurrent alpha and beta ERD following auditory onset, with alpha ERD persisting across the remainder of the trial epoch and beta ERD fading ~850 ms following auditory onset. This pattern of activity is broadly consistent with the tenets of Analysis by Synthesis. In particular, alpha ERS is generally considered a marker of cortical inhibition [86, 87], and is thought to sharpen attention by enabling the reallocation of cognitive resources [88, 89]. Its emergence in perceptual tasks [28, 90, 91] may be interpreted as evidence of Predictive Coding [27, 92, 93], in which prior knowledge tunes and sharpens neural processing toward expected stimulus features [94]. Specifically, articulatory knowledge derived from facial movements imposes motor-based constraints on upcoming sensory processing [95]. This interpretation is consistent both with the activation of anterior sensorimotor regions by visual speech [96, 97] and the influence of early visual information on auditory processing [23, 75, 98]. It should be noted that the theoretical frameworks accounting for the emergence of the McGurk Effect [49, 99] propose multiple distinct stages; a) the influence of visual context, b) incongruence detection, and c) incongruence resolution [49]. It may then be proposed that alpha ERS emerging prior to auditory onset reflects the influence of visual context on sensorimotor decoding of audiovisual speech.

The concurrent alpha and beta ERD which emerged following auditory onset in the current study has been observed in a number of speech perception studies [20, 30, 91, 100], suggesting that it may characterize sensorimotor responses to such tasks. This pattern of activity is consistent with the tenets of Analysis by Synthesis [26], which proposes that a coarse sketch of the incoming stimulus is mapped onto a motor-based articulatory hypothesis in anterior motor regions and is then validated against the full signal in posterior sensory regions [25, 101]. Interactions between these regions are reflected in alpha and beta channels of the mu rhythm [11], with alpha ERD reflecting the inverse model projection of the sensory signal to motor regions, and beta ERD reflecting the forward model projection to sensory regions for comparison against the full signal [22, 30]. It should be noted that the current study did not employ any connectivity metrics able to identify direction of information flow, and interpretations are based on previous associations of alpha and beta ERD with inverse and forward models, respectively [11, 22]. Since any mismatch arising from the comparison between the full signal and the forward model projection is relayed back to anterior motor regions for iterative hypothesis revision and re-testing, paired alpha and beta ERD is expected to persist until the mismatch is resolved and stimuli are identified. Alpha ERD has been observed to persist beyond the cessation of beta ERD in perceptual tasks [102], where this post-stimulus activity has been interpreted as evidence of contextualizing sensory information by drawing on a repository of stored knowledge [103]. Taken together, these findings may be interpreted to suggest that the cessation of beta ERD at ~800 ms following auditory onset represents the termination of Constructivist hypothesis-test-refine loops, constituting a temporal marker for stimulus identification. However, while overall patterns of mu activity are broadly consistent with interpretations through Analysis by Synthesis, to evaluate the manner in which the sensorimotor system supports the integration of discordant sensory signals onto a unified phonological representation, it is essential to consider between-condition differences.

### 4.2 Left hemisphere differences

When ERSP data from Match trials were compared to data from McGurk trials eliciting sensory fusion, differences were observed in two distinct time windows. The early effect was characterized by weaker alpha and beta ERD from ~400–750 ms following auditory onset in McGurk trials. Previous reports of elevated anterior sensorimotor responses to congruent audiovisual stimuli [48, 104] have been interpreted as evidence of synergistic activation elicited by the increased relevance of matching auditory information [105] consistent with pre-existing heuristics [48, 106]. Within the framework of Analysis by Synthesis, it was hypothesized that the visual signal would be mapped onto an articulatory hypothesis via an inverse mapping (alpha), with a forward model transformation (beta) of that articulatory hypothesis compared against the full signal for hypothesis testing [22]. It may be proposed that the elevated early alpha and beta ERD in Match trials reflects the detection of congruence, with auditory signals confirming articulatory hypotheses generated by the visual signal. It should be noted that this notion is consistent with the presence of a similar pattern in Mismatch trials, as there is no indication based on behavioral responses that incongruence was detected (i.e., subjects responded with either the visual or auditory stimulus component). This proposal is additionally consistent with the results of Jenson et al. [30], in which stronger alpha and beta ERD were interpreted as the successful extraction of a phonological form [42] allowing stimuli to be encoded into working memory. Weaker early alpha and beta ERD in McGurk trials is therefore consistent with the notion that a phonological representation has not been successfully identified in this time window, though it remains to be considered how this early difference relates to the later effect.

The stronger alpha and beta ERD present from ~900 ms onward in McGurk trials when compared to Match trials suggests some degree of elevated processing not elicited during audiovisual congruence. Results could be interpreted through the framework of performance updating [107, 108] to suggest that the elevated late ERD in McGurk trials reflects the recalibration of sensorimotor mappings. That is, phoneme boundaries are updated on a trial-by-trial basis in light of the detected incongruence between sensory streams. Such an interpretation is consistent with behavioral findings showing gradual adaptation to [6] and recovery from (i.e., washout) [109] auditory perturbations. However, previous oscillatory investigations of post-hoc internal model updating in perception have reported beta ERS (i.e., beta rebound) [110–112], and it remains unclear how this relates to the late ERD observed in the current study. Additionally, performance updating would be expected to elicit a trial-to-trial shift in the categorical perceptual boundaries between phonemes [113], the consideration of which is beyond both the design and scope of the current study.

When considered within the framework of Analysis by Synthesis, results are consistent with notions of hypothesis-test-refine loops engaging to resolve the discrepancy between auditory and visual streams [24]. These loops support communication between anterior motor and posterior sensory regions [95, 114] via paired forward and inverse models [115, 116], which are reflected in beta and alpha ERD, respectively [11, 22]. Jenson and Saltuklaroglu [29] recently probed the influence of discrepancy resolution processes on sensorimotor activity in a study comparing mu responses to matched and mismatched syllable pairs in speech discrimination. Mismatched pairs were characterized by stronger alpha and beta ERD in the later trial epoch, which was interpreted as evidence of prolonged working memory processing to resolve violated hypotheses arising via Predictive Coding. The presence of a similar pattern of increased late ERD in McGurk trials in the current study may reflect a prolonged phonological encoding phase to resolve sensory mismatches arising from audiovisual divergence and extract a unified phonological representation.

A protracted time course of working memory encoding has been observed in studies employing complex [117] and degraded [118] stimuli, and it may be suggested that a similar process is engaged during resolution of audiovisual discrepancies. This interpretation is consistent with previous reports of increased response latency for McGurk trials [119], though this assertion is made cautiously for multiple reasons. First, increased response latency for McGurk stimuli is not universally reported [120], with variability across studies potentially rooted in the inclusion/absence of McGurk trials not eliciting sensory fusion (i.e., ‘unfused’ McGurk trials). Second, the design of the current study, in which subjects were cued to respond at a given time (i.e., 1500 ms post-stimulus) precludes meaningful interpretation of response latencies. Third, a prolonged encoding phase constitutes a neural process, while response latency differences represent a behavioral effect. While a link between them appears intuitive, they constitute distinct phenomena and the relationship between the two awaits clarification. Thus, while late mu differences are consistent with notions of a prolonged encoding phase to resolve audiovisual discrepancies via internal modeling, further work is necessary to more clearly support this interpretation.

Alternatively, it may be proposed that the observed left hemisphere findings reflect a more basic effect of audiovisual conflict (i.e., incongruence) detection rather than sensorimotor contributions to the integration of divergent sensory streams onto a unified phonological representation. However, findings from the Mismatch condition (in which sensory fusion did not occur) argue against such an interpretation for two primary reasons. First, no differences were observed between Match and Mismatch trials in any time/frequency range despite the presence of convergent and divergent audiovisual streams, respectively. Second, the differences observed between Mismatch and McGurk trials occur in the same time/frequency ranges as those observed between Match and McGurk trials, suggesting that they index a process occurring during McGurk trials only. Based on the observed differences, it may be proposed that audiovisual incongruence alone is insufficient to elicit differential left hemisphere mu activity. That is, differential left mu activity emerges only in the presence of sensory fusion, a finding consistent with the above interpretations through the framework of Analysis by Synthesis. This assertion is made tentatively, though, as the exclusion of ‘unfused’ McGurk trials in the current study limits the ability to clearly associate results with sensory fusion.

### 4.3 Right hemisphere differences

Similar ERSP patterns were observed in the right hemisphere, with early alpha ERS giving rise to concurrent alpha and beta ERD in all conditions following auditory onset. While the overall similarity of activity patterns across hemispheres is consistent with the notion that sensorimotor processes supporting speech are bilateral [121], patterns of between-condition effects differed between hemispheres. Specifically, right hemisphere alpha and beta ERD from ~500–1000 ms was stronger in Match trials than either McGurk or Mismatch trials, with no differences observed between McGurk and Mismatch. Thus, unlike the left hemisphere in which differences only emerged in the presence of sensory fusion (regardless of congruence/incongruence), right hemisphere differences appear exclusively driven by audiovisual congruence/incongruence. This may be interpreted to suggest that the right hemisphere performs a more general function than the left in audiovisual speech perception, providing a confirmation of early articulatory hypotheses only in the presence of perfect congruence between sensory streams. There is precedent for the notion that the right hemisphere provides a coarser level of speech analysis than the left, with Asymmetric Sampling in Time (AST; Poeppel, 2003) suggesting that differences in temporal integration windows underlie the left hemisphere specialization for phonemic processing [122]. While AST is specifically targeted towards auditory regions, the results of the current study may be interpreted to suggest that a similar hemispheric dissociation is present in anterior sensorimotor regions. Specifically, right hemisphere mu activity supports a general confirmation of audiovisual congruence, while left hemisphere mu activity supports the integration of auditory and visual streams at the phonological level. As the between-condition differences persist later in the left hemisphere, results are consistent with the notion that the right hemisphere provides a coarse analysis of congruence/incongruence, relinquishing later, more fine-grained analysis to the speech-specialized left hemisphere [123].

## 5. General discussion

While results from left and right hemispheres have been discussed individually, it remains necessary to consider how they inform regarding sensorimotor contributions to the multi-stage processing hierarchy [49, 99] underlying the elicitation of the McGurk Effect. Contemporary accounts of the McGurk effect propose three distinct processing stages [49]; early influence of visual content, incongruence detection, and incongruence resolution/integration. Early visual influences on sensorimotor processing emerged bilaterally across conditions, and are reflected in the presence of alpha ERS prior to the onset of auditory information. Early alpha ERS has been observed in a number of perceptual studies [20, 28, 91], and is considered a marker of active sensing [124] which sharpens neural processing by inhibiting information inconsistent with expected stimulus features [125]. Its presence in the current study may suggest that early visual information imposes motor-based inhibitory constraints on later stimulus processing [23]. Additionally, following extraction of an articulatory representation from the visual signal, it can then be used for latter stages of congruence/incongruence detection.

Results of the current study suggest that while both left and right hemispheres support the integration of divergent sensory streams, their contributions are distinct. Specifically, stronger right hemisphere alpha and beta ERD only in the presence of audiovisual congruence suggests a coarser level of analysis, in which synergistic activity elicited by congruent sensory streams [105] emerges only in the presence of perfect congruence. In contrast, data from the left hemisphere suggests a more nuanced role in which further processing (possibly via hypothesis-test loops) is employed to support the integration of discordant sensory streams onto a unified phonological representation [30]. A similar functional dissociation has been observed in the visual domain, with the right hemisphere detecting anomalies/incongruence and the left hemisphere engaging in further processing to resolve detected incongruities [126–128], and the results of the current study suggest that a similar hemispheric dissociation is present in the sensorimotor system. To the best of our knowledge, this is the first study to demonstrate a clear left/right hemispheric dissociation between general incongruence detection and resolution in sensorimotor oscillations during audiovisual speech perception.

While the current study employed perceptual tasks, findings also hold relevance for speech production. Specifically, as the internal model transformations reflected by mu oscillations [11, 22] are active during both speech perception and production [20, 129, 130], results may be interpreted to clarify the functional role of sensorimotor processes during speech. However, it is critical to consider how the sensorimotor dynamics revealed in the current study may be mapped onto speech production. During production, sensory hypotheses against which reafference is compared are based on an efference copy (i.e., corollary discharge) of the motor plan [131, 132], with no sensory mismatch anticipated during accurate speech production. As the Match trials in the current study did not elicit any sensory mismatch and were characterized by a short burst of alpha/beta ERD following stimulus presentation, it may be proposed that an abbreviated time course of concurrent alpha and beta ERD constitutes an oscillatory marker of confirmed sensorimotor predictions. This interpretation is consistent with current conceptualizations of mu rhythms [22] as well as findings from speech production studies in which alpha and beta ERD are temporally aligned to the motoric act [21, 133]. A different patterns of results, however, is observed in the absence of sensory congruence.

In perturbations studies, the unimodal alterations to sensory feedback give rise to incongruence (i.e., error) signals which must be resolved, similar to the those arising from audiovisual mismatch in the current study. The presence of reduced right mu activity in both McGurk and Mismatch trials in the current study suggests a right hemisphere contribution to incongruence detection, similar to the tenets of DIVA in which right PMC integrates multimodal sensory error signals [2]. The observed reduction in magnitude of right hemisphere alpha and beta ERD may be interpreted as either the absence of synergistic interaction of sensory streams [105], or as evidence of reduced confidence resulting from failure to confirm initial hypotheses. Interpretations of reduced confidence are tenable given that elevated levels of uncertainty have been associated with reductions to beta activity [112, 134] and weaker responses to sensory feedback signals reflected by alpha activity [135]. Nonetheless, regardless of the underlying cause, right hemisphere sensorimotor contributions to multisensory integration appear consistent with a basic role of incongruence detection in both perception and production.

Left hemisphere results in the current study were tentatively interpreted as evidence of incongruence resolution processes via iterative hypothesis-test loops instantiated in paired forward (beta) and inverse (alpha) models [22], though this assertion is made tentatively as there were insufficient ‘unfused’ McGurk trials in the current study to conclusively associate results with sensory fusion. While sensory mismatches requiring such resolution processes during speech production typically arises from artificial manipulations such as acoustic [6, 136] or tactile [7, 8] perturbations, they may arise naturally in sensorimotor-linked disorders such as stuttering. Stuttering in particular is thought to be associated with abnormal internal modeling [12, 137] and may arise from a noisy comparison between prediction and reafference [138]. This noisy comparison is thought to give rise to inaccurate feedback signals, with attempts to integrate this into ongoing motor planning potentially underlying the articulatory resets (i.e., repetitions) and postural fixations characteristic of the disorder [139, 140]. To clarify the physiology underlying stuttering, it is thus essential to identify the typical sensorimotor dynamics supporting incongruence resolution processes. The results of the current study suggest that examination of right and left hemisphere mu oscillations during the McGurk paradigm may constitute an effective means of probing the integrity of mismatch detection/resolution processes in stuttering and other sensorimotor-linked disorders.

## 6. Limitations

While the current study identified robust effects of audiovisual congruence/incongruence on sensorimotor processing, several limitations ought to be considered. First, not all subjects produced usable mu rhythms that could be localized to accepted generator sites. A reduced proportion of contributing subjects is common in EEG studies [141], and in this study may have arisen from the use of standardized, rather than subject-specific, head models. Second, sensorimotor network activity was inferred from a single metric. While this interpretation is consistent with the theoretical tenets of Analysis by Synthesis [24, 26] and current conceptualizations of alpha and beta channels of the mu rhythm [11, 22], interpretations would be bolstered by analysis of posterior sensorimotor activity as well as the use of connectivity metrics. Third, while the McGurk paradigm and speech perturbation studies both give rise to sensory mismatches, they do so across different sensory modalities. Perturbation studies induce incongruence between auditory and somatosensory streams, while the McGurk paradigm induces incongruence between auditory and visual modalities. Further work is necessary to determine whether the sensorimotor incongruence resolution processes identified in the current study are consistent across modality pairs.

## 7. Conclusions and future directions

Herein we capitalized on the temporal sensitivity of EEG and the parity of sensorimotor processes arising during speech perception and production [22, 130] to probe the sensorimotor processes supporting the integration of discordant sensory streams onto a unified phonological representation. Results were interpreted according to a multi-stage processing hierarchy [49, 99] identifying a previously unreported hemispheric dissociation of mu oscillations. Consistent with previous reports from auditory [122, 142] and visual [126–128] domains, it is proposed that the integration of discordant audiovisual signals is supported by distinct contributions from left and right hemispheres. Specifically, right hemisphere mu activity supports a coarse estimate of multimodal sensory congruence/incongruence, while left hemisphere mu activity supports a more granular phonological analysis. Within both hemispheres an increase in magnitude of alpha and beta ERD may reflect the confirmation of early sensory hypotheses by multimodal sensory congruence, while a protracted time course and attenuated magnitude of alpha and beta ERD in the left hemisphere may support incongruence resolution processes. Clarifying the dynamics of sensorimotor-based incongruence detection and resolution processes has the potential to elucidate the neural processing differences underlying stuttering and other sensorimotor-linked disorders [133, 143]. Results further suggest that the McGurk paradigm holds promise for probing the integrity of multimodal integration processes in clinical and non-clinical populations.
